# COVID-19 disease and malignant cancers: The impact for the* furin* gene expression in susceptibility to SARS-CoV-2

**DOI:** 10.7150/ijbs.63072

**Published:** 2021-09-21

**Authors:** Dabing Li, Xiaoyan Liu, Lianmei Zhang, Jiayue He, Xianmao Chen, Shuguang Liu, Jiewen Fu, Shangyi Fu, Hanchun Chen, Junjiang Fu, Jingliang Cheng

**Affiliations:** 1 Basic Medical School, Southwest Medical University, Luzhou 646000, Sichuan Province, China.; 2 Key Laboratory of Epigenetics and Oncology, the Research Center for Preclinical Medicine, Southwest Medical University, Luzhou 646000, Sichuan Province, China.; 3 Department of Pathology, the Affiliated Huaian No. 1 People's Hospital of Nanjing Medical University, Huai'an 223300, Jiangsu Province, China.; 4 School of Medicine, Baylor College of Medicine, Houston 77030, Texas, USA.; 5 Human Genome Sequencing Center, Baylor College of Medicine, Houston 77030, Texas, USA.; 6 Department of Biochemistry, School of Life Sciences, Central South University, Changsha 410013, Hunan Province, China.; 7 Department of Medical Technology, Faculty of Associated Medical Sciences, Chiang Mai University, Chiang Mai 50200, Thailand.

**Keywords:** COVID-19, furin, malignant cancers, SARS-CoV-2, susceptibility, cordycepin (CD)

## Abstract

Furin is a proprotein convertase that activates different kinds of regulatory proteins, including SARS-CoV-2 spike protein which contains an additional furin-specific cleavage site. It is essential in predicting cancer patients' susceptibility to SARS-CoV-2 and the disease outcomes due to varying furin expressions in tumor tissues. In this study, we analyzed furin's expression, methylation, mutation rate, functional enrichment, survival rate and COVID-19 outcomes in normal and cancer tissues using online databases, and our IHC. As a result, furin presented with biased expression profiles in normal tissues, showing 12.25-fold higher than ACE2 in the lungs. The furin expression in tumors were significantly increased in ESCA and TGCT, and decreased in DLBC and THYM, indicating furin may play critical mechanistic functions in COVID-19 viral entry into cells in these cancer patients. Line with furin over/downexpression, furin promoter hypo-/hyper-methylation may be the regulatory cause of disease and lead to pathogenesis of ESCA and THYM. Furthermore, presence of FURIN-201 isoform with functional domains (P_proprotein, Peptidase_S8 and S8_pro-domain) is highest in all cancer types in comparison to other isoforms, demonstrating its use in tumorigenesis and SARS-Cov-2 entry into tumor tissues. Furin mutation frequency was highest in UCES, and its mutation might elevate ACE2 expression in LUAD and UCEC, reduce ACE2 expression in COAD, elevate HSPA5 expression in PAAD, and elevate TMPRSS2 expression in BRCA. These results showed that furin mutations mostly increased expression of ACE2, HSPA5, and TMPRSS2 in certain cancers, indicating furin mutations might facilitate COVID-19 cell entry in cancer patients. In addition, high expression of furin was significantly inversely correlated with long overall survival (OS) in LGG and correlated with long OS in COAD and KIRC, indicating that it could be used as a favorable prognostic marker for cancer patients' survival. GO and KEGG demonstrated that furin was mostly enriched in genes for metabolic and biosynthetic processes, retinal dehydrogenase activity, tRNA methyltransferase activity, and genes involving COVID-19, further supporting its role in COVID-19 and cancer metabolism. Moreover, Cordycepin (CD) inhibited furin expression in a dosage dependent manner. Altogether, furin's high expression might not only implies increased susceptibility to SARS-CoV-2 and higher severity of COVID-19 symptoms in cancer patients, but also it highlights the need for cancer treatment and therapy during the COVID-19 pandemic. CD might have a potential to develop an anti-SARS-CoV-2 drug through inhibiting furin expression.

## Introduction

Furin, a paired basic amino acid cleaving enzyme, also known as FUR (FES upstream region), PACE (paired basic amino acid cleaving enzyme), PCSK3 (proprotein convertase subtilisin/kexin type 3), and SPC1 (subtilisin-like proprotein convertase 1) (OMIM: 136950), is cytogenetically located at 15q26.1 and was reported as a proprotein convertase that activates different kinds of regulatory proteins in the constitutive exocytic and endocytic pathway 1. Proprotein convertases are proteases that are synthesized as precursor proteins and require limited proteolysis to convert into the mature and bioactive proteins. Hendy *et al*. in 1995 first reported that furin is responsible for the physiologic processing of proparathyroid hormone to parathyroid hormone (PTH) 2; Dubois *et al*. found that a biologically active transforming growth factor β1 (TGFB1) protein was cleaved by furin from pro-TGFB1 3. Knockout of furin in transgenic mice is shown to be embryonic lethal thus, by conditional knocking out furin in T cells, Pesu et al. in 2008 showed that it allowed for normal T-cell development but impaired the regulatory function in effector T cells, which consequently generated less TGFB1 4. Thus, furin may be vital in maintaining peripheral tolerance due to its nonredundant function in TGFB1 production. Furthermore, furin and other proprotein convertases have been widely reported to play proteolytic regulation of cell entry of viruses, including coronaviruses 5, 6.

Coronavirus disease 2019 (COVID-19) has quickly spread since December 2019 and the cases are rising worldwide 7-9. At the end of August of 2021, the number of diagnosed cases worldwide is nearly 214 million and confirmed deaths are over 4.4 million (https://coronavirus.jhu.edu/). The severe acute respiratory syndrome coronavirus 2 (SARS-CoV-2), responsible for COVID-19, contains an additional furin-specific cleavage site, a PRRA insertion at the S1/S2 boundary in the spike protein 10-12. This specific cleavage site has been shown to promote viral infectivity and syncytia formation, but is not included in SARS-CoV or other coronaviruses 11, 13-15, supporting the hypothesis that this enzyme is indispensably involved in SARS-CoV-2 infection and COVID-19 pathogenesis 15. This is further substantiated by data that showed the loss of furin cleavage site weakens SARS-CoV-2 pathogenesis 16, whereas O-glycosylation modulates furin cleavage of the spike protein of SARS-CoV-2 17, demonstrating a critical role for furin in SARS-CoV-2 pathogenesis and prevention. Inhibition of furin level in normal cells might help fight the viral infection, namely that of SARS-CoV-2. As a note, another protease, transmembrane serine protease 2 (TMPRSS2), also facilitates SARS-CoV-2 entry by cleaving and activating viral glycoproteins for viral uptake 18, 19.

As for COVID-19 viral infections, the critical event for the entry of genetic material into the host cell lies in the activation of the spike protein by host proteases to allow binding to host receptors. With the previous points in mind, the usage of furin inhibitors for COVID-19 therapy is urgently needed 20, 21, and the 3C-like protease (3CL^pro^) inhibitors for COVID-19 treatment may be the best option 22.

Since proprotein convertases require proteolysis to convert immature proteins to mature and bioactive proteins. Regulating furin activity disclosed its importance in processing cancer-related substrates and showed that high furin activity promotes tumorigenesis 6, 23. For example, loss of furin in T cells suppresses mammary tumorigenesis of triple-negative breast cancer 24, likely by impairing its substrates proIGF1R and proIR processing 25. Furin also plays a critical role in KRAS and BRAF-related ERK/MAPK pathway activation and tumorigenesis of colorectal cancer 26. Furin's expression and its role in SARS-CoV-2 infected cancer patients is still unclear 6, 27. Thus, it is critical to predict the cancer patients' susceptibility to SARS-CoV-2 infections and the disease outcomes by evaluating furin expression in different cancer tissues. In this study, we conducted expression profile analyses for *furin* in relation to COVID-19 in different types of normal tissues and cancer tissues to determine its potential role as a targeted therapeutic marker 20, 28, 29.

## Materials and Methods

### Online databases for furin expression analysis

The* furin* gene and protein expression of the normal tissues were conducted in the Human Protein Atlas (HPA) (https://www.proteinatlas.org/ENSG00000140564-FURIN/tissue) 30-32. The mRNA levels of *furin* in the HPA for single cell type (https://www.proteinatlas.org/ENSG00000140564-FURIN/celltype) and cell lines (v20.www.proteinatlas.org/ENSG00000140564-FURIN/cell) were also conducted respectively. The *furin* gene and protein expression levels in tumor tissues were evaluated in the HPA (v20.www.proteinatlas.org/ENSG00000140564-FURIN/pathology). The expression levels of the human *angiotensin converting enzyme 2* (*ACE2*) gene in the lung normal tissues and lung cancer tissue were assessed in HPA, (v20.www.proteinatlas.org/ENSG00000140564-ACE2/tissue) and (v20.www.proteinatlas.org/ENSG00000140564-ACE2/pathology) respectively. The *furin* expression in multiple tumor tissues and corresponding normal control tissues through The Cancer Genome Atlas (TCGA) and Genotype-Tissue Expression (GTEx) databases were conducted using Gene Expression Profiling Interactive Analysis (GEPIA 2) (http://gepia2.cancer-pku.cn/#analysis) 33. Multiple gene comparison for *furin* and *TMPRSS2* in tumor tissues and the corresponded TCGA normal and GTEx data were also evaluated using GEPIA 2.

### Immunohistochemistry (IHC), western blotting analysis and semi-quantitative RT-PCR for furin

Immunohistochemistry (IHC) was performed in Chinese lung and breast cancer tissues using the furin antibody 31, 32, 34. Briefly, the de-paraffinized and re-hydrated tissue sections were treated in the 10 μM sodium citrate buffer at 95 °C for 12 minutes for antigen retrieval. The treated tissue slides were further incubated in the solution with 3% H_2_O_2_ for quenching the endogenous peroxidase. After blocking with 5% bovine serum albumin (BSA), the tissue slides were incubated with the primary antibody by overnight at 4 °C (rabbit monoclonal antibodies, 1:200; cat #: ab183495, Cambridge, CB2 0AX, UK) and then the biotin-conjugated secondary antibody (cat #: SP-9000, ZSGB-Bio, CN) for 60 minutes at room temperature. The bound secondary antibody was visualized by sequentially incubating with the Streptavidin-conjugated horseradish peroxidase (HRP) and its substrate diaminobenzidine (ZLI-9017, ZSGB-Bio, CN).

Cordycepin (CD) was purchased from Chengdu Must Bio-Technology Co.Ltd (Chengdu, Sichuan, P. R. China). Western blotting analysis for furin expression was performed in lung cancer cell line H1975 and breast cancer cell line BT549 with CD treatments (0, 10 µm, 20 µm, 40 µm) for 24 hours. The furin antibody for western blotting was used as same as IHC (rabbit monoclonal antibodies, cat #: ab183495, Cambridge, CB2 0AX, UK). The semi-quantitative RT-PCR for *furin* expression was performed in breast cancer cell line BT549 with CD treatments (0, 10 µm, 20 µm, 40 µm) for 24 hours. RT-PCR primers for *furin* were as follows, 5'-tgtggtgtaggtgtggccta-3' (RT-furin-L), 5'-gctgatggacagcgtgtaga-3' (RT-furin-R). GAPDH was served as internal control. All experiments were repeated at three times.

### Homology analysis

Homologs of furin in humans (NP_002560.1 in protein and NM_002569.4 in gene in GenBank, Ensembl ID: ENSG00000140564.10) and others were conducted from the National Center for Biotechnology Information (NCBI) program (https://www.ncbi.nlm.nih.gov/homologene?Db=homologene&Cmd=Retrieve&list_uids=1930) and were previously described 35, 36.

### Analysis of furin isoform

By analyzing GEPIA2 database (http://gepia2.cancer-pku.cn/#isoform) 33, we conducted the large datasets of TCGA and GTEx to determine furin isoform usage/distribution and domain structures in different tumor tissues.

### DNA methylation analysis for *furin* promoter

The DNA methylation status in the *furin* promoter from the multiple tumor patients and the association between the *furin* expression and its promoter methylation in the normal and cancerous tissues, were performed through the DNA methylation interactive visualization database (DNMIVD) (http://119.3.41.228/dnmivd/query_gene/?cancer=ESCA&gene=furin) 37 and UALCAN (http://ualcan.path.uab.edu/index.html) 38.

### Analysis for *furin* mutation and its affections on COVID-19 receptors' expressions

Gene mutation module for *furin*, were conducted by TIMER2.0 (http://timer.comp-genomics.org/), and compared to the gene expression of *furin*, *ACE2*, *HSPA5* and *TMPRSS2*, which are related to furin mutation status in TCGA database, Genes for *furin*, *ACE2*, *HSPA5* and *TMPRSS2* were determined to be important for SARS-CoV-2 infection.

### Survival analysis of furin expressions

Using fragments per kilobase of exon model per million reads mapped (FPKM) of the *furin* expression levels in patients, two expressed groups were classified and the correlations between furin expression and patient overall survival (OS) with the group cutoff at the median were evaluated in multiple cancer cohorts using GEPIA 2 (http://gepia2.cancer-pku.cn/#survival) to create Kaplan-Meier curves 33, 35, 39, 40. Comparison of the survival contribution of *furin*, *TMPRSS2*, and *ACE2* genes in multiple cancer types were estimated using the Mantel-Cox test, which was also conducted by GEPIA.

### Functional enrichment analysis

The data from Gene Ontology (GO), pathway of Kyoto Encyclopedia of Genes and Genomes (KEGG), and diseases/drugs of the genes co-expressed with furin were analyzed through the Enrichr database (https://maayanlab.cloud/Enrichr/enrich) 41. The p-value <0.01 was used as the cut off criterion.

## Results

### The expressions of furin in normal tissues are biased

**Figure 1:**
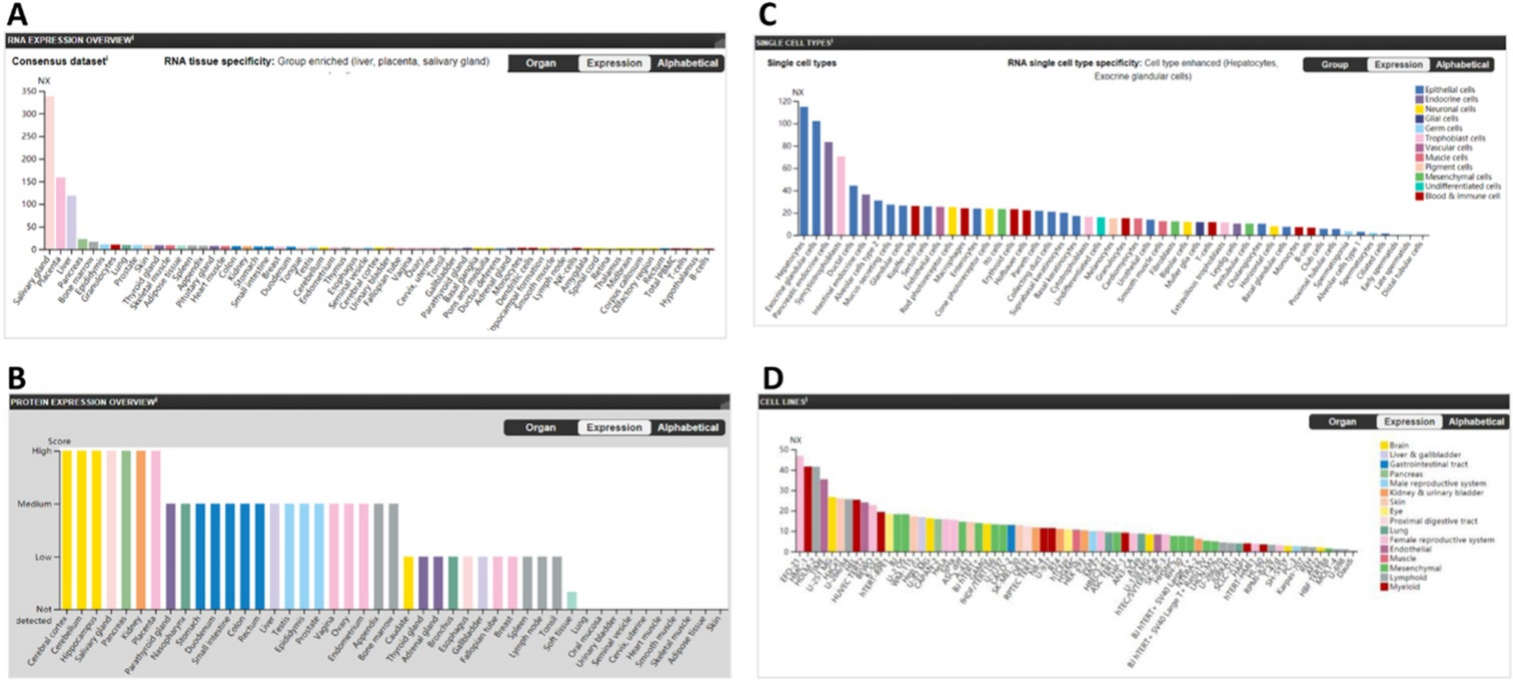
** The furin expression in normal tissues in humans. A.** The *furin* mRNA expression profiles in normal tissues. **B.** The furin protein expression profiles in normal tissues. **C.** The *furin* mRNA expression profiles in normal single cells. **D.** The *furin* mRNA expression profiles in cell lines. NX, consensus normalized expression.

The *furin* mRNA expression profiles were performed from the consensus datasets of HPA, GTEx and FANTOM5. The values for *furin* expression in the salivary gland was found to be highest at 337.9 NX, followed by the placenta (158.9), liver (118.4), pancreas (22.5), bone marrow (19.9); the lungs had the eighth highest expression (9.5). The B-cells was found to be lowest at 1.5 (Figure 1A). The protein levels of furin, based on the scores of high, medium, low, not detected, showed seven tissues with high expression including cerebral cortex, cerebellum, hippocampus, salivary gland, pancreas, kidney, placenta; sixteen tissues with medium expression; eleven tissues with low expression; ten tissues with no detected expression; and one tissue (soft tissue) showed low or no detected expression in different samples (Figure 1B). The expressed *furin* mRNA levels were also presented alongside high expression of protein. Expression profile for the *furin* mRNA in normal single cells showed the hepatocytes had the highest (115), and there was no expression in early spermatids, late spermatids, distal tubular cells, which are germ cells or epithelial cells (Figure 1C). Expression profile for the *furin* mRNA in cell lines showed the EFO-21 had the highest (46.8) and the daudi had the lowest (0.5) (Figure 1D). Thus, these findings revealed biased furin expression profiles for both mRNA and protein in different tissues, cells, and cell lines.

**Figure 2:**
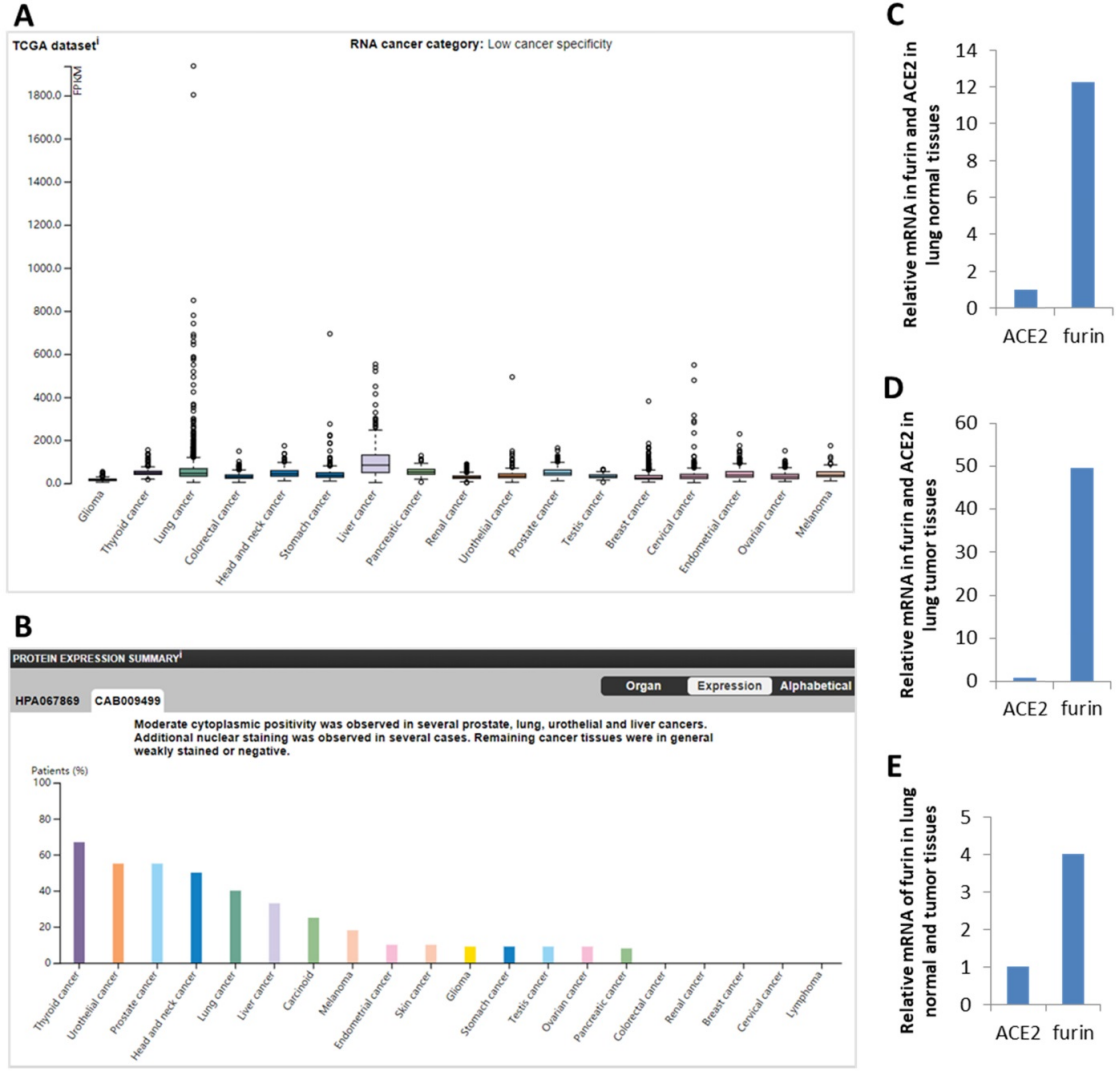
** The furin expressions in cancer tissues in humans. A.** Expression profiles for the *furin mRNA* in cancer tissues. **B.** Expression profiles for the furin protein in cancer tissues. **C.** Relative mRNA levels for *furin* and *ACE2* expression in lung normal tissues. **D.** Relative mRNA levels for *furin* and *ACE2* expression in lung cancer tissues. E. Relative mRNA levels of *furin* in lung normal and tumor tissues in FPKM, Fragments Per Kilobase of exon per Million reads. The p values < 0.01.

ACE2 was reported to be the functional receptor for SARS-CoV-2, playing a crucial role in viral entry into human cells mainly through the lungs 18, 42. Thus, comparison between *furin* and *ACE2* mRNA expression were conducted in normal lungs to reveal possible correlations. We found that *ACE2* expression value is 0.8 NX and *furin* expression value to be 12.25-fold higher (9.5/0.8=12.25) (Figure 2C), showing a correlation between *ACE2* and *furin* expression levels and *furin's* potentially important role in COVID-19 pathogenesis in normal lung tissue.

### The expression results for *furin* in malignant tumor tissues

Expression profile for the furin in cancer tissues from HPA database showed low cancer specificity in mRNA from 17 types of cancers (Figure 2A) and protein from 20 types of cancers (Figure 2B). Specifically, in terms of mRNA levels, liver cancer (LIHC) has the highest with 82.8 FPKM and lung cancer has the fourth with 44.6 FPKM, whereas glioma is lowest with 14.4. In terms of protein levels, thyroid cancer is the highest. Moderate cytoplasmic expression was positivity observed in lung, liver, prostate, and urothelial cancers. Only few nuclear staining was observed in several cases and the remaining cancer tissues were weakly or negative stained (Figure 2B, and data not shown).

Comparison between *ACE2* and *furin* mRNA levels were conducted in 994 samples from the TCGA dataset. We found that *ACE2* levels is 0.9 FPKM and *furin* levels is 44.6 FPKM, and thus levels for furin are 49.56-fold higher than that of *ACE2* (Figure 2D). Further comparison between *furin* levels in normal and cancerous lung tissues found that *furin* levels are 4-fold (49.56/12.25=4.0) increased in cancerous lung tissues than that of the matched normal tissues (Figure 2E). This data implies that furin might play a critical role for SARS-CoV-2 entry and COVID-19 pathogenesis in lungs, particularly in lung cancer tissues.

### The subcellular localizations of furin in the tissues of lung and breast tumor tissues

**Figure 3:**
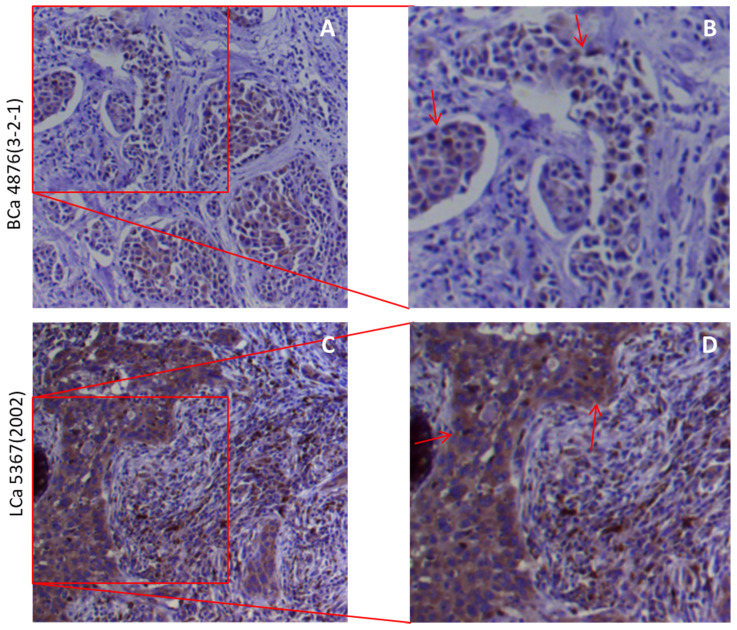
** The furin cellular localization in cancer tissues of the lungs and breast. A, B.** Representative staining for breast cancer patients. A 51-year-old Chinese female of infiltrating ductal carcinoma with tumor size of 3×1.5×1 cm (Her 2+, ER-, PR-). **C, D.** Representative staining for lung cancer patients. A 75-year-old Chinese male of medium-highly differentiated squamous cell carcinoma with tumor size of 2.5×1×0.8 cm (EGFR 2+). BCa, breast cancer, LCa, lung cancer. **B, D.** Enlarged images from A&C respectively.

**Figure 4:**
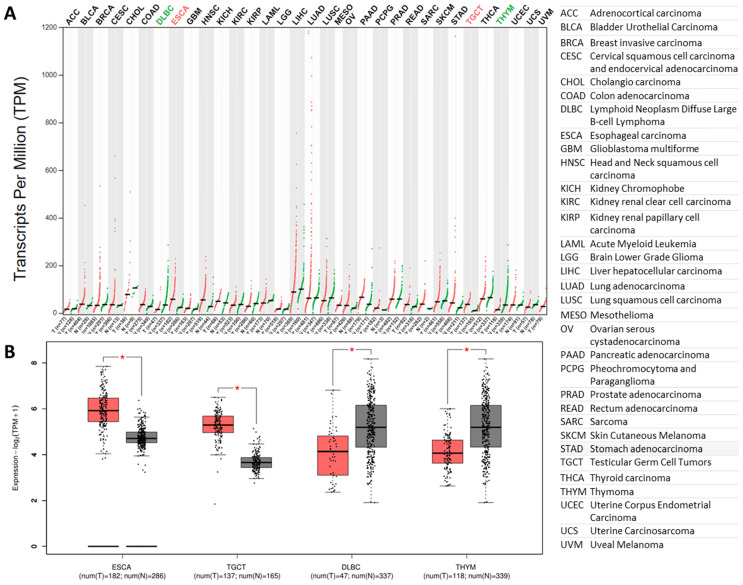
** The *furin* expressions in tumor tissues and matched normal tissues. A.** Expression profiles for *furin* in 33 types of tumor tissues (red files) and the matched normal tissues (green files) (TCGA normal and GTEx data). Tissue-wise expression is used in profiles. Green in DLBC and THYM indicates dowregulated expression whereas red in ESCA and TGCA indicates upregulated expression in tumor tissues compared with the matched normal tissues. **B.** Expression profiles for *furin* in four tumor tissues and their matched normal tissues (TCGA normal and GTEx data) (*: p<0.01). The tumor had the color red, whereas the normal tissue had the color grad. Tissue-wise expression is used in box plots. Right panel shows the full name of cancer types.

The cytoplasmic and membranous sub-localization for receptors, particularly for membranous sub-localization, may facilitate the entry by virus. Thus to determine the spatial distribution of furin at a subcellular level in the human tissues, we did IHC in lung and breast tumor tissues, and the representative results are shown in Figure 4. IHC staining revealed the presence of cytoplasmic and membranous furin signals with moderate intensity in both breast cancer tissues (Figure 3A) and lung cancer tissues (Figure 3B). This cytoplasmic and membranous subcellular localization for furin in human tissues implies furin's possible role in viral infection.

### The expression results for *furin* in malignant tumor tissues and corresponding normal samples

Previous findings showed that furin levels have a 4-fold increase in lung cancer tissues than that of the matched normal tissues (Figure 2E). Thus, we further compared *furin* mRNA profile in tumor samples of 33 types of cancers, including the lungs, and their corresponding normal tissues. The results showed that all types of cancer tissues expressed *furin*, and the highest levels were found in LUAD (Figure 4A). Although most cancer tissues showed an increase of furin (Figure 4A), the* furin* expressions were significantly upregulated only in 2 types of cancers, esophageal carcinoma (ESCA) and testicular germ cell tumors (TGCT) (Figure 4A in red, 4B of left two panels, p<0.01). On the other hand, the *furin* expression levels were significantly decreased in other 2 types of cancers, including lymphoid neoplasm diffuse large B-cell lymphoma (DLBC) and thymoma (THYM) (Figure 4A in green, 4B of right two panels, p<0.01). Altogether, these findings imply that the furin might play a role in SARS-Cov-2 uptake into cells in some tumor tissues of certain cancer patients.

### The results for methylation comparison of *furin* promoter in the tissues of TGCT, DLBC and their matched normal tissues

**Figure 5:**
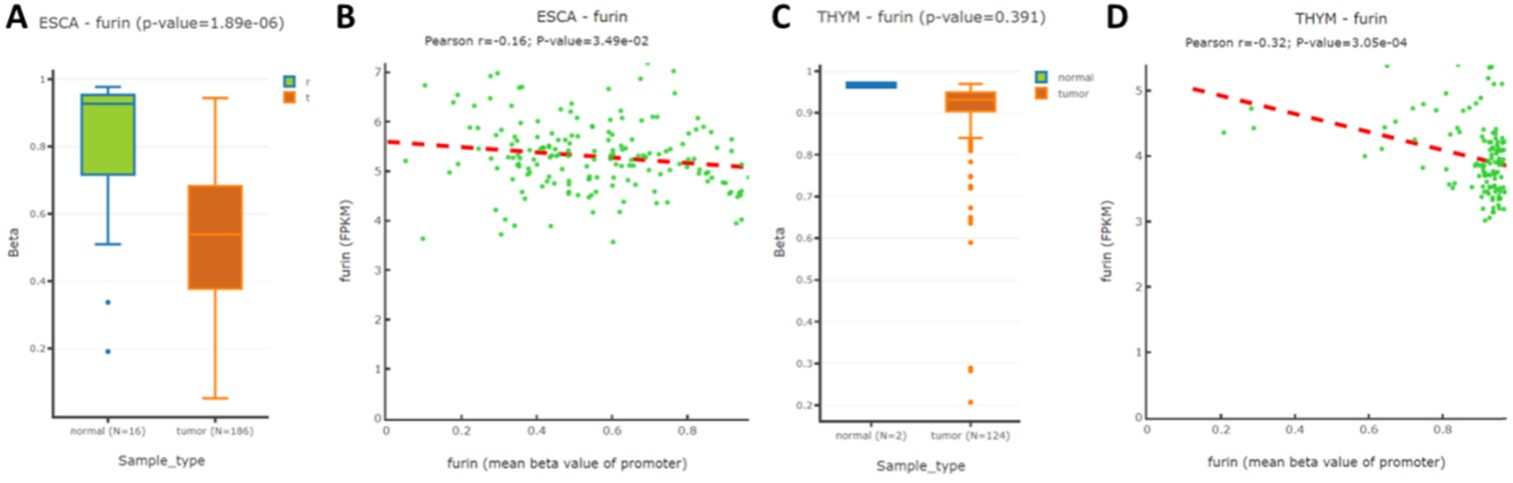
** The *furin* promoter methylation in ESCA and THYM tumor tissues, and their corresponding normal tissues. A.** The promoter methylation for regulating *furin* expression in ESCA. **B.** Pearson analysis for correlation between *furin* mRNA expression and its methylation in ESCA samples. **C.** The methylation of the promoter to regulate *furin* expression in THYM. **D.** Pearson analysis for correlation between *furin* mRNA expression and its methylation in THYM samples. Pearson correlation between methylation of *furin* gene promoter and FPKM was calculated.

To further understand whether methylation modification affects *furin* expression, the TCGA and GEO databases by DNMIVD was applied to evaluate the* furin* promoter methylation in ESCA, TGCT, DLBC, THYM tissues and their matched normal tissues. We analyzed these 4 cancers because furin expressions were significantly changed due to oncogenesis. Although the *furin* promoter methylation in TGCT and DLBC and their matched tissues are currently not available, the promoter methylation statuses in both ESCA and THYM were decreased in comparison to the matched normal tissues respectively (Figure 5A, p≤0.01; 5C, p=0.391), demonstrating *furin* expression and promoter methylation are significantly inversely correlated in ESCA, but not in THYM. Furthermore, using Spearman and Pearson correlations analysis, we disclosed an inverse correlation between the *furin* expression and its promoter methylation for ESCA and THYM tissues (Figure 5B & 5D). Thus, *furin* promoter methylations may be a regulatory mechanism for furin expression in the pathogenesis of ESCA and THYM patients.

### Results for furin isoform usage and its structures in cancer tissues

**Figure 6:**
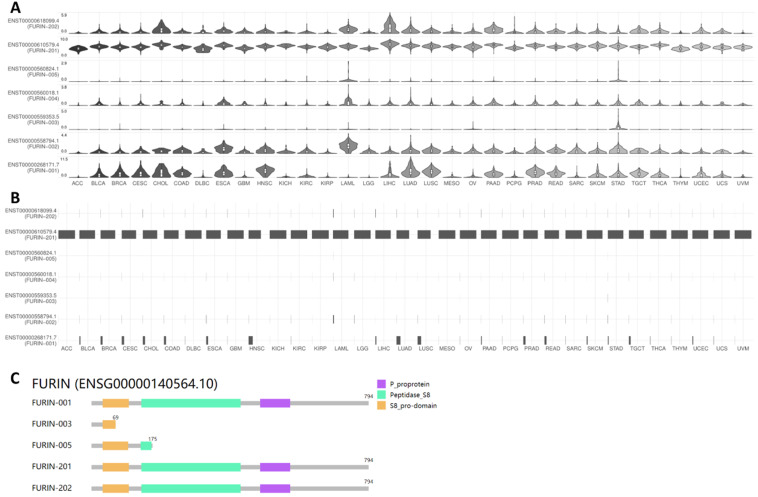
** Furin isoform usage and its structures in different types of cancers. A, B.** Isoform usage for furin. The profiles for the expression distribution (violin plot) are presented in panel A, and isoform usage (bar plot) are presented in panel B. The x axis shows isoforms, and the Y axis shows cancer types. **C.** Isoform structures for furin are also shown. Multiple isoforms and visualized domains are presented in an interactive plot. Please note two isoforms are missing: ENST00000558794.1, ENST00000560018.1.

ACE2 isoforms expressed in the airway epithelium have been reported to differentially contribute to host susceptibility to SARS-CoV-2 43; isoforms of other SARS-CoV-2 receptors or entry proteins might also play similar roles. Thus, we used GEPIA2 database to understand furin isoform prevalence and its structures in 31 types of tumor tissues, and the results are shown in Figure 6. From Figure 6A&B, we noticed that seven isoforms in total are expressed in tumor tissues but with differentially expressed levels (Figure 6A), and isoform ENST00000610579.4 (FURIN-201) usage is the highest in all cancer types, followed by ENST00000268171.7 (FURIN-001) as the second highest; other isoform levels were found to be very low or none (Figure 6B). Further isoform structure prediction showed that FURIN-001, FURIN-201 and FURIN-202 have the domains P_proprotein, Peptidase_S8 and S8_pro-domain, which encoded the same 794 amino acids (Figure 6C). FURIN-005 lacks all of P_proprotein and part of Peptidase_S8, whereas FURIN-003 lacks all of P_proprotein and Peptidase_S8, and part of S8_pro-domain (Figure 6C), demonstrating the functional roles for these isoforms for FURIN-001, FURIN-201 and FURIN-202 in tumorigenesis and SARS-CoV-2 entry into different tumor tissues, particularly FURIN-201.

### Comparisons between furin and TMPRSS2 expression in tumor tissues, and furin conservations among species

**Figure 7:**
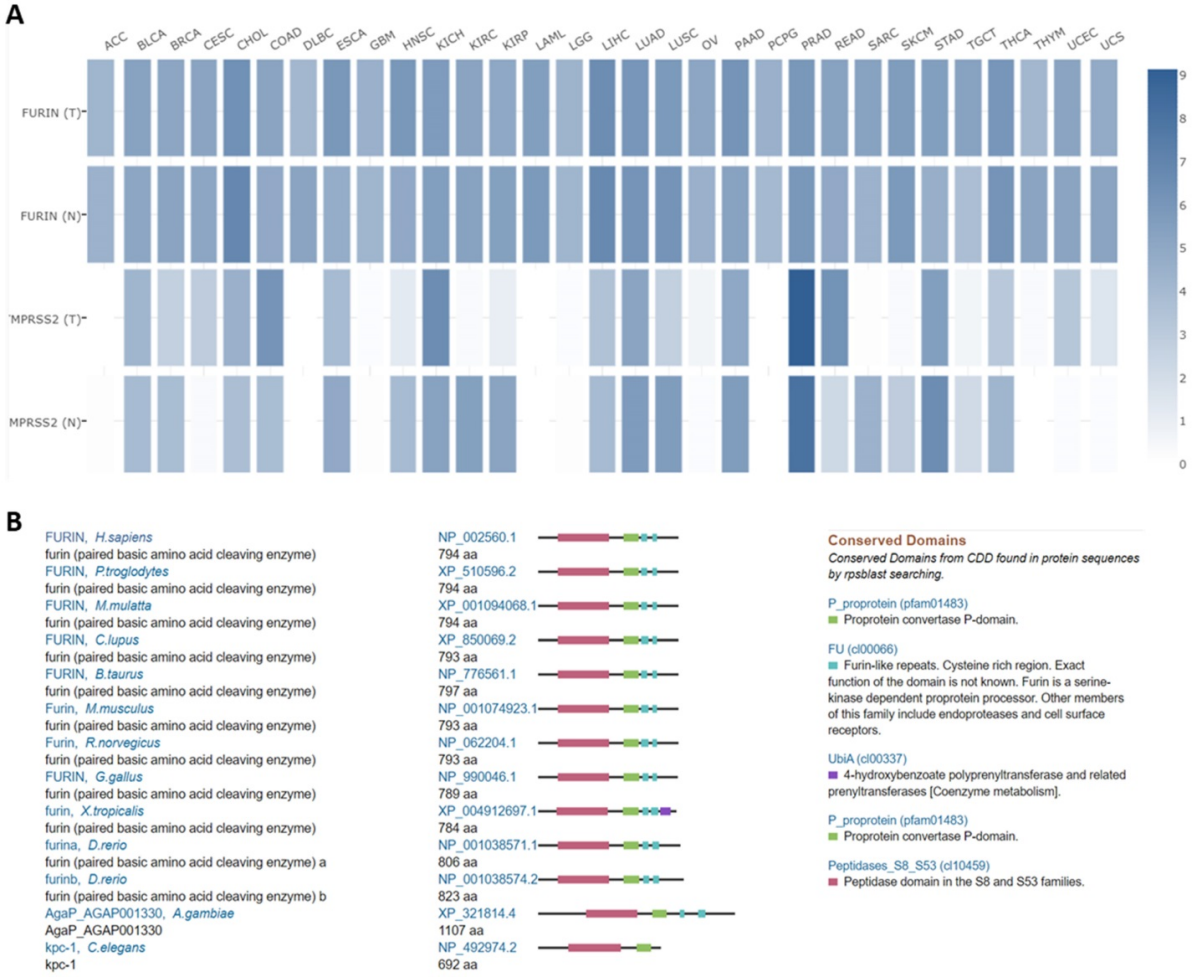
** Expression comparisons between *furin* and *TMPRSS2* in tumors and their corresponding normal TCGA and GTEx data** (**A**). In this panel, an interactive heatmap is used where “T” represents tumor tissues and “N” normal tissues. Homologs and conservations of the furin proteins are presented in thirteen of the different species (**B**).

Both furin and TMPRSS2 are proteinases 2, 3, 44 which are essential for SARS-CoV-2 proteolytic activation 45. Comparisons between furin and TMPRSS2 in different tumor tissues were conducted in TCGA normal datasets, and results are shown in Figure 7A. From Figure 7A, we disclosed that the furin expression is higher than that of TMPRSS2 in most of tumor tissues, except in PRAD, demonstrating that furin might facilitate tumorigenesis and COVID-19 viral entry in most types of cancers.

Homologs and conservation of the furin proteins in thirteen different species also revealed furin is highly conserved, demonstrating it might play a role in COVID-19 viral entry in other species as well (Figure 7B).

### Furin mutation results in cancer tissues and its affection or correlation on ACE2, furin, HSPA5 and TMPRSS2 expressions

**Figure 8:**
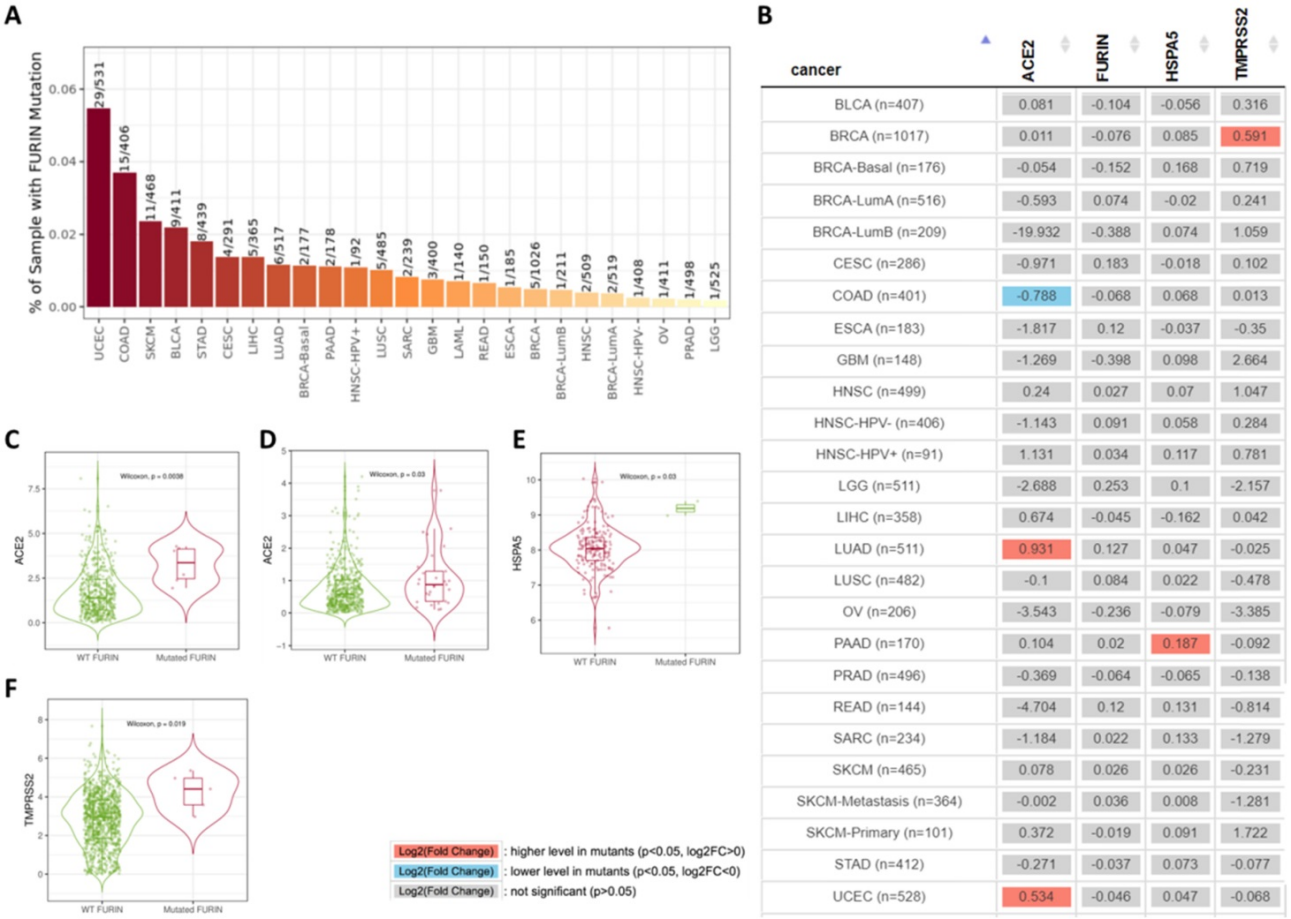
** Furin mutations in multiple cancer types and possible furin's effect on ACE2, HSPA5 and *TMPRSS2* expressions. A.** TIMER2.0 shows a bar plot presenting the furin mutant frequency in the indicated TCGA cancer types. **B.** The heatmap shows the log2 fold expression changes for genes of *ACE2*, *furin*, *HSPA5* and *TMPRSS2* for each cancer type. **C.** Differential *ACE2* expression level in LUAD (sample numbers, n=511). **D.** Differential *ACE2* expression level in UCEC (sample numbers, n=528). **E.** Differential *HSPA*5 expression level in PAAD (sample numbers, n=170). **F.** Differential *TMPRSS2* expression level in BRCA (sample numbers, n=1017).

**Figure 9:**
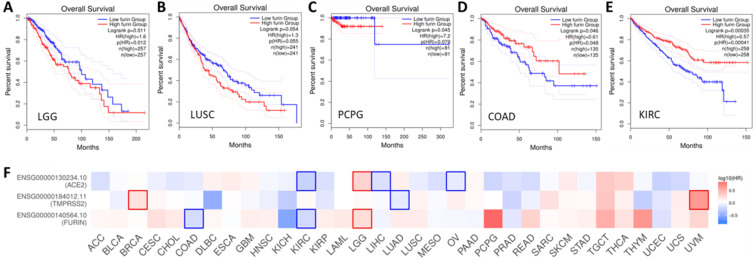
** Overall survival (OS) analysis of *furin* expression from the cancer patients in LGG (A), LUSC (B), PCPG (C), COAD (D) and KIRC (E).** The GENT2 databases were applied to evaluate on the TCGA cohort data and plot Kaplan-Meier curves. F. Comparison among the survival contribution of *furin*, *TMPRSS2*, *ACE2* genes in multiple cancer types, estimated using Mantel-Cox test.

Tumor tissues usually contain gene mutations which causes malignant and recurrent after therapy. Furin mutations in cancers might affected or correlated other receptor expression, thus we conducted by TIMER2.0 analysis to look at the co-relationship between mutant furin and other SARS-CoV-2 receptor proteins. We firstly found that, in 23 types of cancers, UCES has the highest mutant frequency (29/531), whereas LGG had the lowest (1/525) (Figure 8A). Further analysis of the furin mutations' co-relation on ACE2, furin, HSPA5 and TMPRSS2 expression were conducted. We found that furin might elevate ACE2 expression in both LUAD (Figure 9B, C) and UCEC (Figure 9B, D); might reduce ACE2 expression in COAD (Figure 8B); might elevate HSPA5 expression in PAAD (Figure 9B, E); and might elevate TMPRSS2 expression in BRCA (Figure 8B, F). These results indicated that furin mutations might mostly increase expression in ACE2, followed by HSPA5 and TMPRSS2 in some cancers (Figure 8C-F), indicating furin mutations' possible regulatory roles for COVID-19 viral entry in cancers by increase of some receptor's expression. Of course, the patient number with furin mutants are less in PAAD which may hardly draw the solid conclusion. Further study for more samples including mutant patients should be conducted.

### Prognoses for cancer patients based on *furin* expression

Given we found that furin expression is higher in some tumor tissues, clinical relationship between *furin* expression and OS outcomes was conducted. The results are shown in Figure 9, which shows high expression of *furin* is significant inversely correlated with long OS in LGG (Figure 9A, p<0.05), and inversely correlated, though not significantly, with long OS in LUSC (Figure 8B, p=0.055) and PCPG (Figure 9C, p=0.079). High expression of *furin* is significant correlated with long OS in COAD (Figure 9D, p>0.049) and KIRC (Figure 9E, p=0.0004). Thus, furin expression may be a favorable prognostic marker in above five types of cancer patient's survival. Given that furin and TMPRSS2 are both proteinases and ACE2 was reported as the main viral receptor for COVID-19, comparison between OS and TMPRSS2, ACE2 and furin was conducted and found that only KIRC and LGG show the same patterns of OS (Figure 8F), demonstrating their ubiquity in different cancers.

### Function analysis results for co-expressed genes with *furin* in different cancers

**Figure 10:**
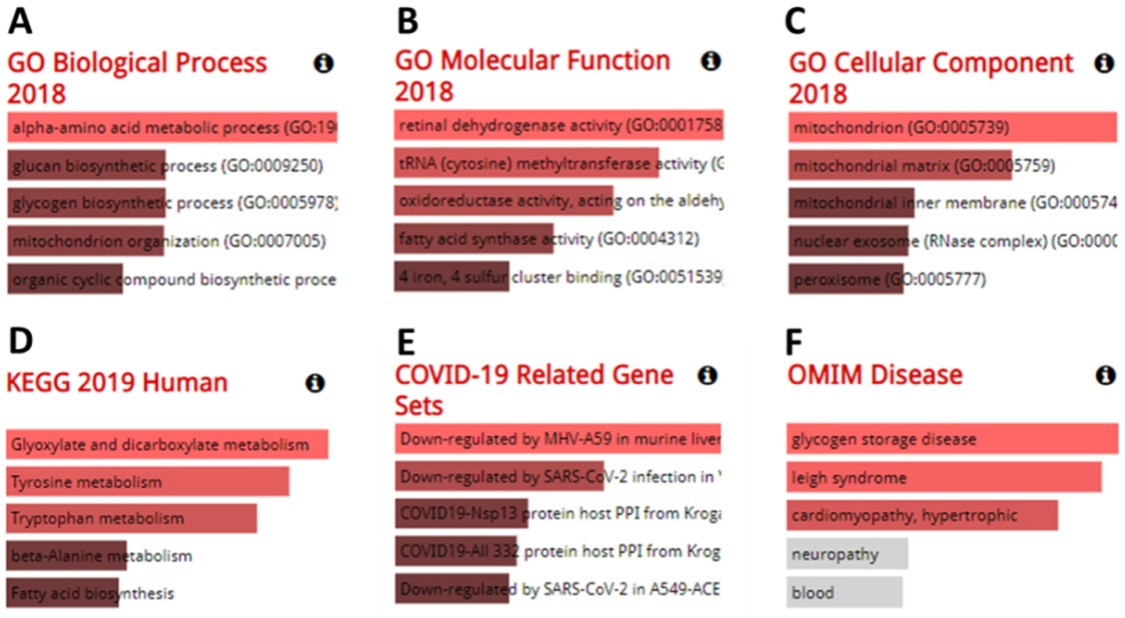
** The results from analysis of GO enrichment and KEGG pathways.** The enriched information for biological processes (**A**), molecular functions (**B**), cellular components (**C**) in analysis of ontologies, KEGG (**D**) in analysis of pathways, COVID-19 related gene sets (**E**), and OMIM disease (**F**) in diseases/drugs, were acquired via the Enrichr database, on the basis of the furin-associated genes. GO, gene ontology; KEGG, kyoto encyclopedia of genes and genomes.

Genes co-expressed with furin were also determined. Co-expression analysis using GEPIA 2 revealed the top 100 genes most similar to *furin* in TCGA tumors, which has a similar expression pattern to other cancer types (Supplementary Table 1). The GO analyses from the Enrichr database (375 genes) are shown in Figure 10A-F. The results showed that we identified the following biological processes, including the alpha-amino acid metabolic process (GO: 1901605), aromatic amino acid family catabolic process (GO: 0009074), glucan biosynthetic process (GO: 0009250), glycogen biosynthetic process (GO: 0005978), kynurenine metabolic process (GO: 0070189), lipoprotein catabolic process (GO: 0042159), mitochondrion organization (GO: 0007005), molybdopterin cofactor biosynthetic process (GO: 0032324), molybdopterin cofactor metabolic process (GO: 0043545), NAD metabolic process (GO: 0019674), NADH metabolic process (GO: 0006734) NADP metabolic process (GO: 0006739), and organic cyclic compound biosynthetic process (GO: 1901362) (Figure 10A, p≤0.01). It also identified the following molecular functions, including retinal dehydrogenase activity (GO: 0001758) and tRNA (cytosine) methyltransferase activity (GO: 0016427) (Figure 10B, p≤0.01); and the following cellular components, including mitochondrion (GO: 0005739), mitochondrial matrix (GO: 0005759), and peroxisomal matrix (GO:0005782) (Figure 10C, p≤0.01). KEGG analysis further elucidated three enriched pathways: glyoxylate and dicarboxylate metabolism, tyrosine metabolism, and tryptophan metabolism (Figure 10D, p≤0.01). Diseases/drugs enrichment analysis found COVID-19-related gene sets, including downregulated by MHV-A59 in murine liver; COVID19-Nsp13 protein host PPI from Krogan, downregulated by SARS-CoV-2 infection in Vero E6 from GSE153940; COVID19-Nsp13 protein host PPI from Krogan (Figure 10E, p≤0.01); and OMIM disease, including glycogen storage disease (Figure 10F, p≤0.0238) and leigh syndrome (Figure 10F, p≤0.0274). Thus, from all this data, we concluded that the furin is mostly enriched in the genes for metabolic and biosynthetic processes, retinal dehydrogenase activity, tRNA methyltransferase activity, and diseases mainly involving COVID-19, demonstrating furin roles for COVID-19 and cancer metabolism.

### Cordycepin (CD) inhibits furin expression in different cancer cells

**Figure 11:**
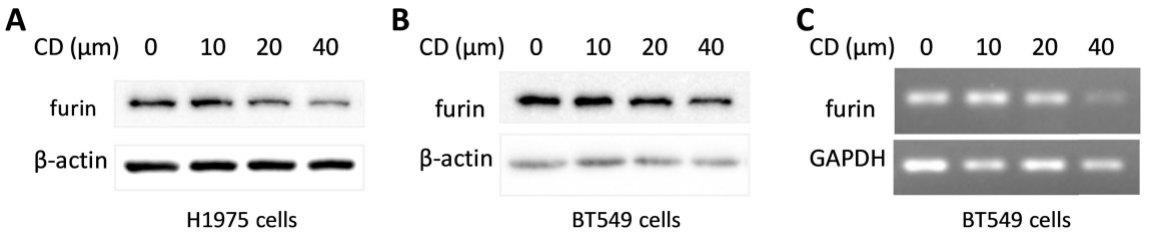
** Cordycepin (CD) inhibits furin expression in different cancer cells. A.** Protein levels in lung cancer cell line H1975. **B.** Protein levels in breast cancer cell line BT549. **C.** RNA levels in breast cancer cell line BT549.

Cordycepin (CD) is one of the most functional components from traditional Chinese medicine (TCM) which showed a broad spectrum of biological activities including anticancer, anti-inflammatory, antidepressant, anti-virial replication, hepato-protective, and neuro-protective properties, etc. 46, 47. To explore the potential in developing CD as an anti- SARS-CoV-2 drug, a furin expression analysis was conducted and the results showed that CD reduced furin expression of protein and/or mRNA in a dosage dependent manner on cancer cell lines H1975 and BT549 (Figure 11). These results implied that CD may have a potential to develop an anti-SARS-CoV-2 drug via inhibiting furin expression.

## Discussion

Patients with cancer are likely more susceptible to viral infection than those without cancer, and consequently more likely to become severely ill or die from COVID-19 48-51. Systematic review analyses showed that COVID-19 patients have more severe malignant cancer onset than that of patients without cancer (33.33% vs 16.09%) 50, 52. Given this discrepancy, it is important to consider the expression of viral entry receptors and proteins in cancer tissues since cancer pathology may affect COVID-19 illness and susceptibility. However, it is unclear whether the patients' susceptibility to SARS-CoV-2 entry and disease severity are correlated with furin expression in different types of cancers.

The SARS-CoV-2 virus, responsible for COVID-19, contains an additional furin cleavage site that should promote viral infectivity, syncytia formation, and cell-cell fusion 10, 11, 53, 54. This site is not presented in SARS-CoV and other coronaviruses 11, 13, 14. Among these convertases, furin could play a predominant role in SARS-CoV-2 invasion. Furthermore, highly expressed SARS-CoV-2 entry receptor or proteins may facilitate viral infection 35, 55-57. The expression levels for furin were also reported to be correlated to neutrophilic inflammation and inflammasome activation 56, 58. Since the pandemic has been continuing at a rapid pace, understanding furin expressions and developing inhibitors as a form of COVID-19 therapy are urgently needed 20, 22. Assessing furin expression in normal and tumor tissues will help foretell cancer patients' susceptibility to SARS-CoV-2 infections and disease outcomes. Even with its potentially vital prognostic and therapeutic use, furin expression and regulation in different malignant tumor tissues, in correlation with patient survival and susceptibility to SARS-CoV-2 entry, were unclear. In this study, using informatics analyzed from online databases, we revealed that *furin* is highly conserved in different species and highly expressed in normal tissues of humans, especially in comparison to other proteases like TMPRSS2 44, 58, and found in the salivary gland, placenta, liver, pancreas. It is also increased in some cancer tissues. *furin* mRNA levels were 49.56-fold higher than that of ACE2 in lung normal tissues and 4-fold higher in lung cancer tissues, implying that SARS-CoV-2 might not only use TMPRSS2 or ACE2 to attack tissues from normal and cancerous organ tissues, but also use furin, particularly in lung cancer tissues. By analyzing the furin isoform expression and structures in 33 types of tissues, we found that furin expressed seven isoforms in different levels; for isoform usage, FURIN-201 is the highest in all cancer types. Furthermore, isoform structure prediction found that FURIN-001, FURIN-201 and FURIN-202 have the domains P_proprotein, Peptidase_S8, and S8_pro-domain, demonstrating their functional roles in tumorigenesis and SARS-CoV-2 infection in different tumor tissues. The GO and KEGG analyses found that the furin is mostly enriched in genes for metabolic and biosynthetic processes, retinal dehydrogenase activity, tRNA methyltransferase activity, and diseases mainly including COVID-19, demonstrating furin's roles in COVID-19 pathogenesis and cancer metabolism. Moreover, high *furin* expression is inversely correlated with long OS in LGG, LUSC and PCPG. Altogether, increased furin expression in cancer patients, such as LGG, LUSC and PCPG, might play a critical role in the susceptibility to SARS-CoV-2 uptake and severity of COVID-19 clinical symptoms.

The promoter methylation statuses in both ESCA and THYM were decreased in comparison to those of normal tissues, demonstrating *furin* expression and promoter methylation are significantly inversely correlated in ESCA, but not in THYM. Spearman and Pearson correlations disclosed an inverse correlation between *furin* expression and its promoter's methylation in ESCA and THYM tissues. Thus, *furin* promoter methylation may be one of the regulatory mechanisms for furin overexpression in ESCA tumors and pathogenesis. Tumor tissues usually have gene mutations which lead to recurrent malignancies. We found that UCES has the highest mutant frequency (29/531). Effect on *ACE2*, *furin*, *HSPA6* and *TMPRSS2* expression due furin mutation revealed that *furin* might elevate *ACE*2 expression in LUAD and UCEC, might reduce *ACE2* expression in COAD, might elevate *HSPA5* expression in PAAD, and might elevate *TMPRSS2* expression in BRCA. These results indicated that *furin* mutations correlated with expression of *ACE2*, *HSPA6* and *TMPRSS2* in some cancers, implying furin's possible regulatory roles for the expression regulation of viral entry proteins.

Moreover, CD showed a broad spectrum of biological activities including anti-virial replication, anticancer, anti-inflammatory, antidepressant, hepato-protective, and neuro-protective properties 46, 47. The recently study revealed that CD has a strong binding affinity with S protein and Mpro proteins of SARS-CoV-2 by molecular docking, and anti-SARS-CoV-2 testing further showed CD's antiviral action *in vitro* 59. Our furin expression analysis showed that CD reduced its expression in a dosage dependent manner, further supporting the possibility to develop an anti-SARS-CoV-2 drug. This also implies CD's role for anti-SARS-CoV-2 by inhibiting furin expression.

## Conclusions

In conclusion, *furin* is highly expressed in normal tissues and increased significantly in some tumor tumors, implying the possibly higher susceptibility to SARS-CoV-2 uptake and possibly high disease severity of COVID-19 clinical symptoms for cancer patients. Our studies highlight the value of combating cancers by targeting therapeutic strategies for furin in the COVID-19 pandemic. CD might have a potential to develop an anti-SARS-CoV-2 drug through furin inhibition.

## Supplementary Material

Supplementary table.
